# Empowering Healthcare through Precision Medicine: Unveiling the Nexus of Social Factors and Trust

**DOI:** 10.3390/healthcare11243177

**Published:** 2023-12-15

**Authors:** Bian Ted Nicholas Tan, Md. Irfanuzzaman Khan, Md. Abu Saleh, Dawa Wangchuk, Md. Jakir Hasan Talukder, Catherine R. Kinght-Agarwal

**Affiliations:** 1 Canberra Business School, University of Canberra, Canberra 2617, Australia; u3218749@uni.canberra.edu.au (B.T.N.T.); abu.saleh@canberra.edu.au (M.A.S.); dawa.wangchuk@canberra.edu.au (D.W.); 2 Advance Computing and Information Science, University of South Australia, Mawson Lakes, Adelaide 5095, Australia; jakirhasan@mkt.jnu.ac.bd; 3 Faculty of Health, University of Canberra, Canberra 2617, Australia; cathy.knight-agarwal@canberra.edu.au

**Keywords:** precision medicine, healthcare technology, UTAUT, social influence, trust in doctors

## Abstract

This study investigated the impact of social factors on the acceptance of precision medicine (PM) using a quantitative survey grounded in the Unified Theory of Acceptance and Use of Technology (UTAUT) framework. The findings revealed that social influence has a significantly positive effect on PM acceptance, while the influence of social media is found to be insignificant. Performance expectancy emerged as the most influential factor, demonstrating a significant relationship with PM acceptance. Trust plays a crucial moderating role, mitigating the impact of social factors on PM acceptance. While exploring the mediating effects of trust, we identified a significant mediation effect for social influence and performance expectancy on PM acceptance. However, the mediation effect of social media influence is insignificant. These findings highlight the importance of trust in shaping decisions regarding PM acceptance. These findings have significant implications for healthcare practitioners and policymakers aiming to promote the adoption of precision medicine in clinical practice.

## 1. Introduction

Precision medicine (PM) is an approach to disease management that considers an individual’s medical and genetic information that will allow doctors and researchers to predict more accurately which treatment and prevention strategies for a particular disease will work in which groups of people [1,2]. The concept of PM commonly evolves around personalized treatment strategies. It places a strong emphasis on the process and the utilization of data, particularly drawing from clinical and other relevant information. This approach involves categorizing patients into sub-groups based on comprehensive data analysis, ultimately enabling more accurate and tailored therapeutic solutions [3]. It contrasts with a one-size-fits-all approach, in which disease treatment and prevention strategies are developed for the average person, with less consideration for the differences between individuals. PM essentially aims to optimize health outcomes by using knowledge about genomics, lifestyle, and environment factors to better tailor medicine and healthcare and stratifies patients into sub-groups to enable more precise diagnoses and targeted therapies [4]. The aim of PM is to improve clinical outcomes for individual patients and minimize unnecessary side effects for those less likely to have a response to a particular treatment by providing clinicians with new tools, knowledge, and therapies to select which treatments will work best for which patients [5].

The recent surge of PM has primarily been fueled by rapid advancements in genomic medicine. The sequencing of an individual’s genetic code has paved the way for precision healthcare, innovative therapies, and diagnostics and brought about revolutionary changes in various domains, such as public health, cancer care, and the handling of rare diseases [6]. According to Konig and colleagues (2017), the term “PM” should not be perceived merely as the culmination of a novel patient stratification with clinical utility. Instead, it should be regarded as a highly sophisticated and complex process. Within this process, stratified medicine and tailored therapy can emerge as interim outcomes, and the results should be looped back to accumulate knowledge, thereby enhancing precision [3]. It is imperative to maintain appropriate messaging and actions that align with these expectations in order to achieve success [7]. However, gaps still remain at the social, clinical, organization, and policy levels when it comes to the adoption of PM [6,8]. While advancements in the broader applications of PM have been facilitated by the emergence of extensive databases, it may also create uncertainty for both healthcare professionals and patients. Thus, it is important to address ethical, legal, and social challenges in fostering trust between patients and providers [9].

This study particularly focuses on the social factors that influence patient acceptance of PM technology as an integral part of their healthcare. Extant research indicates that the adoption of innovative practices such as PM often progresses slowly and unevenly, with socially advantaged groups typically experiencing quicker benefits compared to disadvantaged groups, including racial and ethnic minorities, low-income populations, and uninsured patients [7,10]. A recent study on Hispanics/Latinos from diverse countries in Latin America has shown that families play a pivotal role in healthcare decision-making, and obtaining input from family members is deemed essential before undergoing a precision medicine (PM) genetic test or treatment [7]. Moreover, trust in healthcare providers along with individual, familial, and societal benefits have also been identified as facilitators of PM uptake [7]. Building on the above, this study aims to examine the impact of the social and individual factors likely to influence PM acceptance. In the healthcare domain, there are several societal factors that are shaping healthcare considerations amongst individuals. By recognizing the issues that influence the user’s decision on a particular technology, healthcare providers can predict, pre-empt, and prepare for the possible outcomes to make the adoption a success and minimize risks of discontent and distrust [11]. Moreover, there is an increasing recognition of the necessity for adopting a sociotechnical approach in implementing new Health Information Technology (HIT) which underlines the significance of giving attention not only to the technical components of a system but also to its social aspects during implementation [12].

The results of this study also contribute to the generic technology adoption literature by providing important insights into the factors influencing PM acceptance from the perspective of consumers or patients. To be precise, this study examines the roles played by social media, social influence, performance expectancy, and trust in shaping the decision to accept PM.

### 1.1. Theoretical Background

This study is grounded on The Unified Theory of Acceptance and Use of Technology (UTAUT) [13]. UTAUT is a widely used framework in the domain of generic technology acceptance, which aims to explain user intentions to use an information system and continue to expand the usage behavior. This model was created to draw more attention to the acceptance process compared to previous individual models by merging eight separate models that had their origins in psychology, sociology, and communications. These models are the Theory of Reasoned Action (TRA); the Technology Acceptance Model (TAM); the Motivational Model; the Theory of Planned Behavior (TPB); a model combining the TAM and the TPB; the model of PC Utilization; the Innovation Diffusion theory, and the Social Cognitive theory. Each model was created to predict and explain user behavior via a variety of independent variables and the UTAUT model was conceived to merge conceptual and empirical similarities across these eight separate models [13,14]. In the UTAUT, four (4) key constructs (performance expectancy, effort expectancy, social influence, and facilitating conditions) are direct determinants of usage intention and behavior. The key constructs are defined as follows: 1. performance expectancy (PE) “is the degree to which an individual believes that using the system will help him or her to attain gains in job performance”; 2. effort expectancy (EE): “is the degree of ease associated with use of the system”; 3. social influence (SI): “is the degree to which an individual perceives that [it is] important others believe he or she should use the new system”; 4. facilitating conditions (FC): “is the degree to which an individual believes that an organizational and technical infrastructure exists to support use of the system” [13].

**Figure 1:**
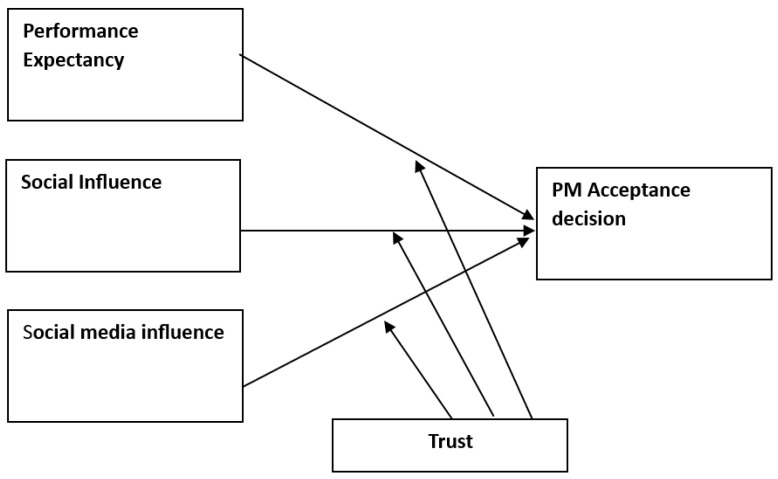
Conceptual model.

In our proposed theoretical model, we have suggested some modifications to the UTAUT to fit the context of PM acceptance (see Figure 1). In our adaptation, we have replaced the construct of facilitating conditions with social media influence, as we believe this factor plays a pivotal role in influencing user intentions and behaviors related to technology acceptance [15]. The decision to exclude effort expectancy from our model is grounded in a thoughtful consideration of the specific contextual factors inherent in PM technology use. Effort expectancy, characterized as the degree of ease associated with using a specific technology or system [13] is a conventional dimension in technology acceptance models, emphasizing user-friendliness and simplicity [16]. However, the distinctive characteristics of PM informed our choice to exclude this dimension. PM is primarily managed by healthcare providers rather than end-users [9,17,18]. The complexity of PM involves the integration of genomic, environmental, digital health, and patient-reported data, with healthcare providers possessing the technical expertise for navigation and use [9,19]. Thus, the notion of ‘ease of use’ is not relevant in this case, as user-friendly interfaces are unlikely to be the primary concern for patients when it comes to PM technology acceptance.

### 1.2. Hypothesis Development

As stated earlier, PM represents a paradigm shift in healthcare [20], promising personalized treatment strategies tailored to individual genetic, environmental, and lifestyle factors [21]. Understanding the drivers of PM acceptance is paramount in realizing its transformative potential. In this study, we propose a conceptual model that integrates key determinants, including performance expectancy, social influence, and the impact of social media. Additionally, we examine the moderating role of trust in doctors, acknowledging the pivotal influence of healthcare providers in shaping patient decisions regarding PM.

### 1.3. Performance Expectancy

Performance expectancy, grounded in the UTAUT model, posits that individuals are more likely to accept and use a technology if they perceive it as beneficial and effective [13,14]. The concept builds on the fundamental premise that users are motivated to adopt technology when they anticipate positive and valuable outcomes from its use [13,14]. In the context of PM, individuals who perceive it as offering substantial improvements in healthcare outcomes and treatment efficacy are more likely to embrace this innovative approach [22]. Previous research has consistently demonstrated the significant impact of performance expectancy on technology adoption decisions in the healthcare domain [23,24]. We posit that individuals’ perceptions of the benefits and effectiveness offered by PM will play a crucial role in shaping their decisions to accept and adopt this innovative approach. As users evaluate the potential positive impact on healthcare outcomes and treatment efficacy, their inclination to accept PM into their healthcare decisions is expected to be positively influenced. Thus, we propose the following:

**H1.**  *Performance expectancy affects the PM acceptance decision*.

### 1.4. Social Influence

Social influence emphasizes the role of subjective norms and the influence of significant others on technology acceptance [13]. In the context of PM, the opinions and recommendations of peers, family members, and healthcare professionals can exert a powerful sway on an individual’s decision-making process. Empirical studies in healthcare technology adoption have consistently demonstrated the influential role of social influence in shaping acceptance decisions [25,26,27]. These studies consistently demonstrate that individuals are not isolated decision-makers in the adoption of healthcare technologies; rather, their choices are dependent on the social context that surrounds them. Thus, we posit the following hypothesis:

**H2.**  *Social influence affects the PM acceptance decision*.

### 1.5. Social Media Influences

The rise of social media platforms has transformed information dissemination and opinion-sharing, making them influential channels for technology-related decision-making [28]. Since its inception, social media’s popularity has grown and it has been widely adopted by users for more convenient and widespread sharing of images, videos and comments with regards to many topics and issues [29], including health, and it provides alternative resources for health-information seekers [30,31]. In healthcare, social media platforms serve as dynamic spaces for individuals to access diverse perspectives, discussions, and recommendations [32]. For example, Patients Like Me provides a comprehensive social media platform that includes online health profiles, patient details, and medical histories, along with interactive features enabling members to exchange detailed reports. Site users have the opportunity to engage in various disease-specific communities, enabling them to access information tailored to their specific healthcare requirements [33]. The constant information exposure in different social media platforms leads to information processing, ultimately influencing attitudes and behavioral intentions [34].Thus, the following hypothesis has been proposed:

**H3.**  *Social media channels positively influence the PM acceptance decision*.

### 1.6. Trust as a Moderator

Trust in healthcare providers is a critical factor in patient decision-making, influencing acceptance of new healthcare interventions [35,36]. We posit that trust in doctors may moderate the relationship between antecedents (performance expectancy, social influence, and social media influence) and the PM acceptance decision. Specifically, individuals with higher levels of trust in their healthcare providers may be more influenced by the recommendations and opinions of healthcare professionals [37], potentially amplifying the impact of social influence and performance expectancy on their PM acceptance decision. Thus, we propose:

**H4a.**  *Trust moderates the relationship between performance expectancy and the PM acceptance decision such that higher trust results in a higher influence of PE on the PM acceptance decision*.

**H4b.**  *Trust moderates the relationship between social influence and the PM acceptance decision such that higher trust results in a lower effect of SI on the PM acceptance decision*.

**H4c.**  *Trust moderates the relationship between social media influence and the PM acceptance decision such that higher trust results in a lower effect of SMI on the PM acceptance decision*.

### 1.7. Trust as a Mediator

The extant technology adoption literature suggests that the role of trust is fundamentally salient in the acceptance and adoption of innovative technologies [38]. As individuals engage with complex and personalized medical solutions, their level of trust in the system and its components becomes a pivotal factor influencing their acceptance of such technologies [39]. More, trust is multifaceted, encompassing aspects of benevolence, openness, reliability, competence, and honesty [40]. While prior research has confirmed the mediating role of trust in diverse contexts [41,42], it is still not clear how trust mediates the relationship between influential factors and the acceptance of PM. Thus, we posit the following hypotheses:

**H5a.**  *Performance expectancy positively influences trust in precision medicine*.

**H5b.**  *Social influence positively influences trust in precision medicine*.

**H5c.**  *Social media influence positively influences trust in precision medicine*.

**H5d.**  *Trust positively influences precision medicine acceptance*.

**H5e.**  *Trust mediates the relationship between performance expectancy and PM acceptance*.

**H5f.**  *Trust mediates the relationship between social media influence and PM acceptance*.

**H5g.**  *Trust mediates the relationship between social influence and PM acceptance.*

**Figure 2:**
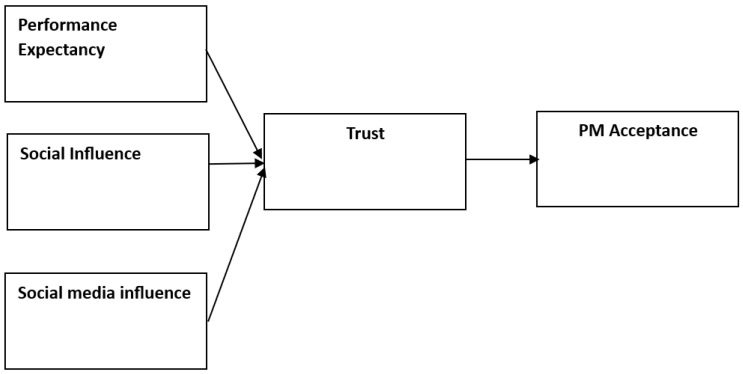
Alternative model.

The above comprehensive models offer valuable insights into the multifaceted determinants of PM acceptance, bridging the gap between technology acceptance theories and healthcare decision-making processes. By examining the interplay of performance expectancy, social influence, social media influence, and trust, this study contributes to a deeper understanding of the factors driving the acceptance of PM, ultimately informing strategies to enhance its implementation and uptake in clinical practice (see alternative model in Figure 2). 

## 2. Methods

This study used a cross-sectional design and collected data from Singapore residents through a geo-targeted online survey distributed across various social media channels. The choice of a quantitative survey method aligns with previous research in a similar field, which utilized this approach to assess the influence of independent variables on dependent variables [43]. A total of 471 individuals participated, yielding 377 valid responses, resulting in an 80% response rate. A total of 81% of the respondents are within the age range of 26–35, whereas 19% are above 36 years old. Participation in the survey was voluntary, and every effort was made to uphold the autonomy, data integrity, and confidentiality of the respondents throughout the research process.

### Measures

The measures for this are adopted from extant research and contextualized accordingly. For example, the measures for trust were based on the work of Lysaght and colleagues [4]; performance expectancy [44] and social influence [45,46] are originally inspired from the work of Venkatesh and colleagues [13] and later adopted by others. The measures for social media influence were inspired by the work of Iftikhar and Abaalkhail [47] and Wang et al. [46]. Additionally, we ensured the reliability and validity of the measurements by confirming that all alpha reliabilities exceed 0.7, and the AVE for each construct is equal to or higher than 0.5 [48]. Table 1 outlines the bivariate correlation matrix of constructs used in this study, which demonstrates that all hypothesized relationships are positively correlated, indicating a consistent pattern of association among the variables.

## 3. Results

This study used structural equation modeling (SEM) to investigate the proposed hypotheses. SEM is a statistical tool that enables researchers to examine relationships between independent variables (IVs) and dependent variables (DVs) [49]. The assessment of the model fit demonstrated that the measurement model aligns well with the established criteria indicating a satisfactory fit ( χ^2^/DF = 2.73; IFI = 0.97; TLI = 0.95; CFI = 0.97; RMSEA = 0.069. R^2^ = 0.587) [50]. The results of the SEM analysis are provided in Table 2.

The findings from the SEM analysis were then used to evaluate the research hypotheses. The analysis revealed that the influence of social media on the acceptance of PM was positive but statistically insignificant (*p* > 0.05). This suggests that social media influence may not strongly impact the acceptance of precision medicine. On the other hand, social influence yields a significant positive relationship with PM acceptance, with a regression coefficient of 0.250 (*p* < 0.001). This indicates that individuals who perceive greater social influence are more likely to accept precision medicine. The critical ratio (C.R. = 4.319) further supports the strength of this relationship. Furthermore, performance expectancy shows a significant association with PM acceptance, established by a substantial coefficient of 0.716 (*p* < 0.001). This implies that individuals who have higher expectations regarding the effectiveness of precision medicine are more likely to accept it.

The interaction effect between trust and social media influence emerges as statistically significant (*p* < 0.05). This finding suggests that heightened trust offsets the positive influence of social media on PM acceptance. In other words, individuals with greater trust in healthcare providers exhibit less susceptibility to social media’s impact on their acceptance of PM. Similarly, those who place higher trust in their healthcare providers are less swayed by social influence in their adoption of precision medicine (*p* < 0.05).

However, it is noteworthy that trust does not act as a moderating factor in the relationship between performance expectancy and PM acceptance. This could be attributed to the notion that individuals’ perceived utility of PM is predominantly shaped by external factors like promotional efforts or subjective knowledge, wherein trust may not play a pivotal role in determining the perceived benefits. Additionally, factors such as one’s knowledge about PM may yield a substantial moderating influence on the relationship between performance expectancy and PM acceptance, as highlighted in prior research [46]. Overall, our findings explain the pivotal role of trust as a potent moderator, exerting noticeable influence in the relationship between PM acceptance and its antecedents.

While examining the role of trust as a mediator in influencing individuals’ acceptance of PM (Table 3), we find positive and significant relationships between social media influence and trust (*p* < 0.05), highlighting the impact of online platforms on building trust. Similarly, social influence significantly contributes to trust (*p* < 0.01), emphasizing the role of peer pressure in shaping trust. Performance expectancy also significantly influences trust (*p* < 0.01). Building upon these relationships, trust emerges as a key mediator, significantly influencing PM acceptance (β = 0.357, *p* < 0.01). While, exploring indirect effects through trust, we identified significant mediation effect for social influence (β = 0.117, *p* < 0.01) and performance expectancy (β = 0.057, *p* < 0.05) on PM acceptance. However, the mediation effect of social media influence is insignificant (β = 0.042, *p* > 0.05). These findings highlight the crucial role of trust as a mediator in shaping the acceptance of PM, offering practical insights for those involved in healthcare decision-making.

## 4. Discussion

Our results indicate that both social influence and performance expectancy have considerable influence on the PM acceptance decision, which is consistent with prior research in the healthcare domain [51,52]. Specifically, individuals who perceive higher social influence and hold greater expectations about the performance of PM are more likely to accept it. The interaction between trust and social influence suggests that individuals with higher levels of trust in doctors may be less susceptible to external social pressures when making decisions about precision medicine. This implies that a strong doctor–patient relationship, characterized by trust, may serve as a protective factor against undue influence from social circles. Thus, our findings highlight the importance of fostering trust between healthcare providers and patients in the context of PM adoption. Prior studies also showed that both trust and communication were positively related to patient satisfaction and the perceived quality of health care services [53,54]. Establishing and maintaining a strong doctor–patient relationship may not only enhance patient satisfaction [53] but also likely positively influence their acceptance of cutting-edge medical interventions.

However, it is noteworthy that social media influence does not seem to exert a statistically significant impact on PM acceptance. The non-significant impact of social media influence is an interesting finding and calls for further inquiry, suggesting that while social media serves as a platform for information [55,56], individuals may not regard this information as highly influential in their decision-making process regarding precision medicine. Moreover, the diverse and potentially conflicting nature of information on social media may contribute to this non-significant impact, highlighting the complexity of factors influencing acceptance decisions in this context.

In terms of the moderating effect, trust moderates the relationship between social media and PM acceptance, as well as social influence and PM acceptance. This implies that individuals with greater trust in their healthcare providers were less influenced by social media and their social circles in their acceptance of PM. However, trust did not moderate the relationship between performance expectancy and PM acceptance, indicating that other external factors may play a more substantial role in shaping the performance expectations of individuals. Overall, the findings highlight the significant role of trust in the acceptance of PM [57].

In terms of the mediating effect of trust, the significant relationships identified between social media influence, social influence, and performance expectancy and trust confirms the multifaceted nature of trust formation in the context of PM. These results emphasize the potential of online platforms and social networks in fostering trust, and the importance of addressing individuals’ expectations regarding the performance of PM solutions. Moreover, the role of trust as a mediator in influencing PM acceptance is an important finding and deserves in-depth investigation. Our findings demonstrate the need for healthcare providers and policymakers to prioritize interventions that enhance trust, considering its direct and indirect impact on PM acceptance.

### 4.1. Theoretical Implications

This research significantly contributes to the broader field of technology acceptance by shedding light on the pivotal role of trust in doctors as a critical factor in shaping individuals’ acceptance of PM. While prior studies in technology acceptance have often examined the basic tenants of the UTAUT and the UTAUT2 model [58,59,60], this study goes beyond by demonstrating that trust moderates the impact of social influence and social media influence on PM acceptance. In doing so, it expands our understanding of the important role of social factors and trust in the acceptance of advanced medical technologies like PM. Moreover, the confirmation of trust as a mediator in the pathway to PM acceptance adds a significant layer to existing theoretical frameworks. Additionally, the varying significance of indirect effects through trust suggests a need for targeted interventions. While social influence and performance expectancy exhibit significant mediation effects, social media influence does not. Thus, it is important to tailor communication strategies to address specific influential factors that may optimize the impact of interventions on PM acceptance.

### 4.2. Practical Implications

The interaction between trust and social influence suggests that individuals with higher levels of trust in doctors may be less susceptible to external social pressures when making decisions about precision medicine. This implies that a strong doctor–patient relationship, characterized by trust, may serve as a protective factor against undue influence from social circles. Thus, our findings highlight the importance of fostering trust between healthcare providers and patients in the context of precision medicine adoption. Prior studies also showed that both trust and communication were positively related to patient satisfaction and the perceived quality of health care services [53,54]. Establishing and maintaining a strong doctor–patient relationship may not only enhance patient satisfaction [53] but also likely positively influence their acceptance of cutting-edge medical interventions.

The above findings also point towards the importance of considering the patient–provider relationship as a key determinant in the successful implementation of innovative medical interventions. Extant research consistently indicates that patients who have trust in their doctors tend to exhibit positive behaviors such as adhering to treatment plans and maintaining continuity in their care [61]. Indeed, individuals who have greater trust in their healthcare providers tend to describe participating in more beneficial health-related practices [62]. This adds to the body of technology adoption as empirical studies pertaining to the consumer’s attitude towards PM are relatively scarce [19,63]. PM relies on the data and information from participants who have to be willing to offer their personal information on the promise that that information will result in knowledge to improve human health, so from that perspective the under-representation of potential users is a concern because it cannot be assumed that people everywhere perceive the issues and prioritize values in the same way [4].

## 5. Conclusions

This study emphasizes the critical role of trust in the successful implementation of PM initiatives, urging policymakers and healthcare institutions to prioritize strategies aimed at strengthening trust between healthcare providers and patients. Beyond the immediate implications, our findings point towards promising avenues for future research, particularly in understanding the complex nexus between trust and social factors within the realm of the PM acceptance decision. Subsequent studies may focus on designing and assessing interventions aimed at bolstering trust within healthcare settings, thereby fostering a conducive environment for the acceptance of PM. Future research endeavors can also focus on practical interventions that can be implemented to strengthen trust between patients and healthcare providers and facilitate the seamless incorporation of PM into routine healthcare delivery. While the findings focus on the importance of trust, social factors, and the PM acceptance decision, this study is not without limitations. A limitation of this study is the relatively small percentage of participants over the age of 36. This may impact the generalizability of the findings, as acceptance of PM might vary significantly among older cohorts, warranting caution in extending this study’s conclusions to broader age groups. Moreover, this study relies on cross-sectional data, and the rapidly evolving nature of healthcare technologies and patient perceptions may impact the generalizability of the findings. Future research could use longitudinal designs to capture the dynamics of trust-building and the evolution of PM adoption over time, providing a more comprehensive understanding of how various factors influence the adoption of PM.

## Figures and Tables

**Table 1:** Correlation matrix.

	1	2	3	4	5
Performance expectancy	1				
Social Influence	0.311 **	1			
Social media influence	0.118 *	0.510 **	1		
Trust	0.276 **	0.438 **	0.304 **	1	
PM Acceptance	0.676 **	0.468 **	0.289 **	0.357 **	1

Notes: Correlation is significant at the 0.05 * and 0.01 **.

**Table 2:** Hypotheses testing (Model 1).

Path			Estimate	S.E.	C.R.	*p*
Social Media Influence	→	PM Acceptance	0.036	0.033	1.088	0.27
Social Influence	→	PM Acceptance	0.250	0.058	4.319	***
Performance expectancy	→	PM Acceptance	0.716	0.065	10.938	***
Trust x Performance Expectancy	→	PM Acceptance	−0.0523	0.0565	−0.9260	0.35
Trust x Social Media Influence	→	PM Acceptance	−0.123	0.0416	−2.9632	0.00
Trust x Social Influence	→	PM Acceptance	−0.0977	0.0441	−2.2145	0.02

*** *p* < 0.01.

**Table 3:** Hypothesis testing (Model 2).

Path			Estimate	S.E.	C.R.	*p*
Social Media Influence	→	Trust	0.118	0.028	2.160	0.031
Social Influence	→	Trust	0.328	0.040	5.763	***
Performance expectancy	→	Trust	0.160	0.040	3.256	***
Trust	→	PM Acceptance	0.357	0.065	7.211	***
Indirect effect						
Social Media Influence > PM Acceptance (Indirect Effect through Trust)			0.042	0.015	2.81	0.242
Social Influence > PM Acceptance (Indirect Effect through Trust)			0.117	0.012	9.76	***
Performance Expectancy > PM Acceptance (Indirect Effect through Trust)			0.057	0.018	3.17	0.016

*** *p* < 0.01.

## Data Availability

Data will be made available upon reasonable request.
